# Neoadjuvant Down-Sizing of Hilar Cholangiocarcinoma with Photodynamic Therapy—Long-Term Outcome of a Phase II Pilot Study †

**DOI:** 10.3390/ijms161125978

**Published:** 2015-11-06

**Authors:** Andrej Wagner, Marcus Wiedmann, Andrea Tannapfel, Christian Mayr, Tobias Kiesslich, Gernot W. Wolkersdörfer, Frieder Berr, Johann Hauss, Helmut Witzigmann

**Affiliations:** 1 Department of Medicine I, Paracelsus Medical University/Salzburger Landeskliniken (SALK), Muellner Hauptstrasse 48, 5020 Salzburg, Austria; and.wagner@salk.at (A.W.); ch.mayr@salk.at (C.M.); t.kiesslich@salk.at (T.K.); g.wolkersdoerfer@salk.at (G.W.W.); 2 Department of Internal Medicine I, St. Mary’s Hospital, Gallwitzallee 123-143, 12249 Berlin, Germany; wiedmann@marienkrankenhaus-berlin.de; 3 Institute of Pathology, Ruhr-University Bochum, Bürkle-de-la-Camp-Platz 1, 44789 Bochum, Germany; andrea.tannapfel@rub.de; 4 Institute of Physiology and Pathophysiology, Paracelsus Medical University, Strubergasse 22, 5020 Salzburg, Austria; 5 Second Department of Surgery, University of Leipzig, Liebigstraße 20, 04103 Leipzig, Germany; johann.hauss@uniklinik-leipzig.de; 6 Department for General and Visceral Surgery, Städtisches Krankenhaus Dresden-Friedrichstadt, Friedrichstraße 41, 01067 Dresden, Germany; helmut.witzigmann@khdf.de

**Keywords:** bile duct cancer, PDT, sodium porfimer, neoadjuvant therapy

## Abstract

Hilar cholangiocarcinoma (CC) is non-resectable in the majority of patients often due to intrahepatic extension along bile duct branches/segments, and even after complete resection (R0) recurrence can be as high as 70%. Photodynamic therapy (PDT) is an established palliative local tumor ablative treatment for non-resectable hilar CC. We report the long-term outcome of curative resection (R0) performed after neoadjuvant PDT for downsizing of tumor margins in seven patients (median age 59 years) with initially non-resectable hilar CC. Photofrin^®^ was injected intravenously 24–48 h before laser light irradiation of the tumor stenoses and the adjacent bile duct segments. Major resective surgery was done with curative intention six weeks after PDT. All seven patients had been curatively (R0) resected and there were no undue early or late complications for the neoadjuvant PDT and surgery. Six of seven patients died from tumor recurrence at a median of 3.2 years after resection, the five-year survival rate was 43%. These results are comparable with published data for patients resected R0 without pre-treatment, indicating that neoadjuvant PDT is feasible and could improve overall survival of patients considered non-curatively resectable because of initial tumor extension in bile duct branches/segments—however, this concept needs to be validated in a larger trial.

## 1. Introduction

Bile duct cancer (cholangiocarcinoma, CC) is a rare tumor with an incidence of about 3–5 per 100,000 in Western Europe and shows perihilar extension in nearly two thirds of cases [1,2,3]. The chance for curative resection of hilar CC is limited by the extent of perihilar tumor spread along the bile duct branches/segments and/or involvement of portal venous or hepatic arterial branches. Most patients become symptomatic at an advanced stage of perihilar tumor extension, and therefore hilar CC is resectable for cure only in the minority of patients (approx. 30%). Resection techniques have considerably improved due to en-bloc resection combined with extended liver resection and/or portal vein resection, and in selected cases with pancreatoduodenectomy (PD) and/or liver transplantation (OLT). Optimized tumor imaging, indication criteria, and surgical techniques have improved five-year survival rates up to 50% after curative resection of hilar CC [4,5,6]. Nevertheless, current surgical strategies provide R0 resection in only 60%–78% of patients considered to be resectable and tumor-free margins tend to be short, which may be partly explained by the fact that even during surgery, tumor extension is often difficult to define [7,8,9]. However, even after complete (R0) resection, recurrence rate can be as high as 50%–76%, in particular at the anastomosis or in the liver [10,11,12].

As palliative anti-tumor therapy, photodynamic therapy (PDT) using hematoporphyrin derivative (Photofrin^®^) and laser light irradiation achieved reliable local tumor ablation in non-resectable hilar CC, improved palliation, and prolonged survival time at a tolerable low rate of adverse events [13,14,15,16,17,18,19,20,21]. Prospective trials confirmed prolonged survival time of combined PDT and biliary drainage as compared with biliary drainage alone [14,21,22]. The efficacy of PDT could even be improved, which has been demonstrated for next generation photosensitizers, such as talaporphin sodium or temoporfin. Their utilization showed promising results in recent clinical trials due to a deeper tumoricidal effect at a low rate of side effects [23,24,25]. In an adjuvant setting, PDT has shown beneficial effects in a clinical series in CC patients with local recurrence after surgery [26]. PDT as a neoadjuvant approach may decrease the rate of tumor recurrence by local selective ablation of CC and dysplastic epithelium beyond the feasible resection margins, thus down-sizing the primary tumor and purging peritumoral bile ducts from tumor cell nests and dysplastic epithelium prior to resection and bilioenteric anastomosis. In a phase II pilot study, we have shown the feasibility of neoadjuvant PDT for downsizing and extended curative (R0) resection of hilar CC in all seven patients considered a priori non-resectable because of advanced local extension of CC along the bile duct branches/segments [27]. PDT using hematoporphyrin derivative (Photofrin^®^) had led to destruction of the superficial 4 mm layer of the bile duct cancer and showed high tumor selectivity in the resected bile duct specimens. There were two local recurrences and the 1-year recurrence free survival rate was 83% in this pilot trial [27,28].

We now analyzed the long-term outcome (follow-up > 15 years) of this pilot study cohort (*n* = 7, [27]) in order to explore if neodjuvant PDT for down-sizing of CC and purging of tumor margins prior to surgery for intended curative resection might improve the long-term outcome of a priori non-resectable patients. In a case control manner we compared these survival data with that of a historical control cohort of 35 patients with hilar CC we had resected curatively (R0) without pretreatment [20].

## 2. Results 

### 2.1. Clinical Protocol and Short-Term Results

Seven patients (median age 59 (43–72) years, 6 men, 1 woman) with hilar CC either Bismuth type II with intrapancreatic extension (*n* = 1) or with local extension considered non-R0-resectable (Bismuth type IV, *n* = 3; Bismuth type III, *n* = 3) gave informed consent to receive PDT prior to resective surgery (comp. Table 1). The phase II pilot study—approved by the Ethics Committee of the University of Leipzig—recruited from January 1997 to November 2001. Patients, methods, surgery, pathohistological data and short-term follow-up (median 16 (9–40) months) of the pilot series have previously reported in detail [27,28]. For PDT, hematoporphyrin derivative (Photofrin^®^ 2 mg/kg body weight) was injected intravenously 24–48 h before laser light irradiation (wavelength 630 nm, 240 Joules/cm length) of the tumor stenoses and adjacent bile duct segments (2–2.5 cm) proximal and distal to the tumor margins as detected by MRI as well as by ERC with intraductal ultrasound. All seven patients had been bridged from PDT to surgery by insertion of biliary endoprostheses to drain both liver lobes. Within 5 days before surgery, all patients were reevaluated by MRI scan and ERC with exchange of endoprostheses to check for visible extent of PDT-induced tumor necroses and to exclude overt cholangitis. Resection for curative intention was decided by an interdisciplinary board (surgical/endoscopic/radiological). Major resective surgery was performed with curative intention 30–72 days after PDT. In another patient (No. 2, 56 years old, ASA grade I) an advanced Bismuth type IV tumor and liver fibrosis was found during the first laparotomy 4 weeks after neoadjuvant PDT. After a waiting time of 10 months (308 days after PDT), he underwent a combined hepatectomy, PD and OLT procedure. Anastomosis at bile duct segments pretreated with PDT was feasible without undue complications. Tumor extension, pathologic staging, and grading were definitively classified in the en-bloc resected specimen. Surgical procedures, clinical outcome and histopathologic staging were previously described [27,28] and are briefly summarized in Table 2. 

**Table 1:** Clinical data for the seven consecutive patients undergoing neoadjuvant photodynamic therapy (PDT).

Patient	Age/Gender	Bismuth Stage ^1^	UICC Stage ^2^
1	71/male	II (L 0.5 cm, C 3 cm)	II
2	56/male	IV (L 3 cm, R 1.5 cm, C 3 cm)	III
3	52/male	IV (L 4.5 cm, R 1.5 cm, C 2 cm)	IVA
4	72/male	IIIa (L 0.5 cm, R 1.5 cm, C 2 cm)	IVA
5	43/female	IIIa (L 2 cm, R 4 cm, C 1 cm)	III ^3^
6	61/male	IIIb (L 3.8 cm, R 0.5 cm, C 2.2 cm)	IVA
7	59/male	IV (L 1.5 cm, R 3.5 cm, C 2 cm)	IVA

L: left hepatic duct; C: common hepatic duct; R: right hepatic duct. ^1^ Diagnosed by endoscopic retrograde cholangiography, magnetic resonance cholangiography, and intraoperative finding; ^2^ UICC, 2009 [29]; ^3^ this patient had undergone exploratoy laparotomy with proof of a single CC-positive LN in hepatoduodenal ligament in another hospital before referral to PDT. Right hemihepatectomy after neoadjuvant PDT revealed pT1bN0M0V0L0 (Table 2). Reprinted by permission of John Wiley & Sons, Inc. [27].

### 2.2. Long-Term Results

During the follow-up period (16 years), six patients had died, and one patient (No. 2) is alive without CC recurrence 16.0 years after liver transplantation. He is on low-dose immunosuppressive therapy with sirolimus and has compensated cardiac failure (NYHA II-III) 4 months after PTCA with coronary stent insertion for myocardial infarction. The overall 1-, 3-, and 5-year patient survival after surgery was 86%, 57%, and 43%, respectively. Six patients died from tumor recurrence 0.9–7.1 (median 3.2) years after resection. A median disease free survival of 3.1 (range 0.5–6.8) years could be observed. Two patients had a local recurrence (one at the pancreatic margin after PD, one in regional lymph nodes) and four recurred in distant metastases. Follow-up and causes of death are summarized in Table 2.

**Table 2:** Surgical procedures, histopathological staging and follow-up.

Patient	Surgery	Resection	Pathological TNM Stage	Grading	Complications	Tumor Recurrence	Survival (Months)	
							Overall ^1^	Disease-Free
1	Hilar resection, PD	R0	pT2N0M0V0L1	G2		Local	35	19
2	OLT, PD	R0	pT2N1M0V0L1 ^2^	G2	Insufficiency of the pancreatic anastomosis	(None)	>180 ^2^	>180 ^2^
3	Left hemi-hepatectomy (S1–4)	R0	pT3N2M0V1L1	G2		Liver	11	6
4	Right hemi-hepatectomy (S5–8)	R0	pT3N1M0V0L0	G2	Subdiaphragmatic hematoma	Peritoneum	85	81
5	Right hemi-hepatectomy (S5–8) ^3^	R0	pT1bN0M0V0L0	G1		Liver, right Pleura	75	57
6	Left hemi-hepatectomy (S1–4)	R0	pT3N0M0V1L0	G2	Bile leakage	Peritoneum	16	10
7	Right trisegment-ectomy (S1, 4–8)	R0	pT3N0M0V0L1	G2	Bile leakage	Local, Lung	41	37

^1^ after surgery; ^2^ patient alive without recurrence >16 years after surgery. Hepatectomy & PD (at OLT) had revealed a single CC pos. LN in the specimen; ^3^ including resection of right hepatic artery and portal vein; OLT: liver transplantation; PD: partial pancreatoduodenectomy; S: segments. Reprinted by permission of John Wiley & Sons, Inc. [27].

### 2.3. Historical Group Comparison

**Figure 1:**
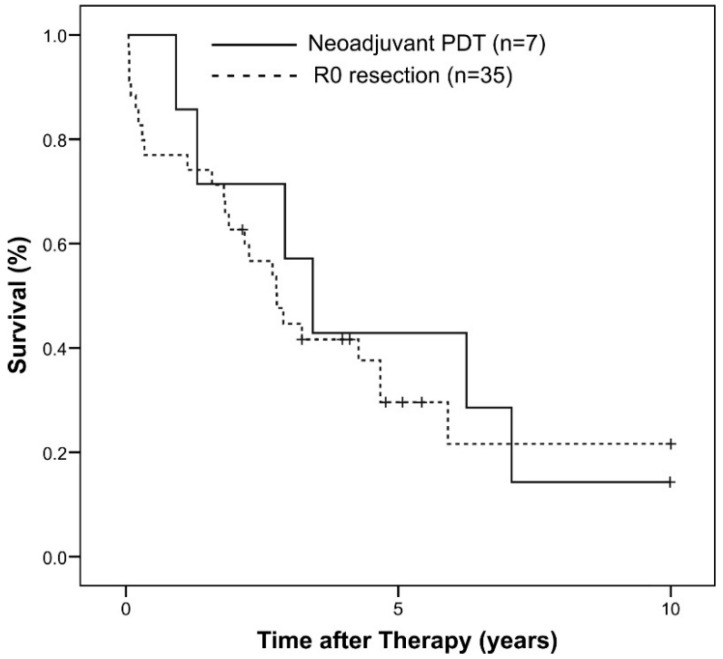
Kaplan-Meier estimation of patient survival: The survival curve of the patients resected R0 after neoadjuvant PDT (*n* = 7) compared with the survival curve reported for the historical cohort of patients (*n* = 35) resected R0 without pre-treatment ([20], *p* = 0.34, log rank test). Individual patients still alive during follow-up are indicated by marks on the curves. Reprinted by permission of Wolters Kluwer Health [20]—promotional and commercial use of the material in print, digital or mobile device format is prohibited without the permission from the publisher Wolters Kluwer Health.

Most characteristics of the historical cohort of patients recruited from 1994 to 2004 [20] and this pilot trial were similar—such as tumor stage, age of patients and laboratory parameters, except for a higher percentage of lymph node-positive patients (43% *vs.* 14%, not significant) and higher Ca 19-9 levels in the current neoadjuvant series as compared to the historical control of R0 resected hilar CC (Table 3). Cholestasis prior to surgery was less severe (after PDT and stenting) in the patients of the neoadjuvant trial. Median overall survival was 3.2 (0.9–7.1) in the neoadjuvant *vs.* 1.8 (0.0–10.6) years in the historical cohort resected R0 (not significant), and longer than 1.25 (0.2–3) years (*p* < 0.05) in historical controls (*n* = 11) that had been resected R1 (compare figure 2 in [20]). The survival curve of the patients resected R0 after neoadjuvant PDT (*n* = 7) compared favorably for seven years with the survival curve reported for the historical cohort of patients (*n* = 35) resected R0 without pre-treatment ([20], *p* = 0.34, log rank test, Figure 1).

**Table 3:** Comparison with data on non-pretreated R0 resections of a historical cohort.

Baseline Characteristics	Neoadjuvant PDT	No Pretreatment ^1^	*p*
Patients (n)	7	35	-
Age (years) ^2^	59 (43–72)	63 (38–78)	0.41
Serum Bilirubin (mg/dL) ^3,4^	1.6 ± 0.5	4.7 ± 1.3	0.47
Ca 19-9 (U/L) ^3^	1347 ± 871	216 ± 66	0.17
Staging			
UICC Stages I & II	29%	26%	0.92
UICC Stage III	14%	3%	0.64
UICC Stage IVA	57%	71%	0.57
Bismuth Type I & II	14%	9%	0.82
Bismuth Type III	43%	49%	0.82
Bismuth Type IV	43%	43%	1.00
Lymph Node-Positive	43%	14%	0.22
Follow-Up			
Overall Survival (years) ^2^	3.2 (0.9–7.1)	1.8 (0.0–10.6)	0.34
1-Year Survival Rate	86%	74%	-
3-Year Survival Rate	57%	41%	-
5-Year Survival Rate	43%	29%	-

^1^ historical control data of 35 previously patients with hilar CC, that had been curatively resected [20]. Comparison performed using Mann-Whitney’s U test or log rank test (survival data); ^2^ median (range); ^3^ mean ± SEM, before surgery; ^4^ after PDT.

### 2.4. Discussion

Surgical resection and OLT are the only potentially curative treatment options for patients with hilar CC. This study presents the long-term results of a cohort of patients considered non-resectable at baseline because of extended tumor involvement of the perihilar bile duct branches/segments [27,28]. After neoadjuvant PDT, curative resection could be performed in all of them. There were no undue early nor late complications of neoadjuvant PDT and surgery, in particular no treatment-related early (30-day) mortality, and no late strictures at the PDT-pretreated biliodigestive anastomoses [27,28]. The long-term outcome of this pilot study compares well to 5-year survival rates after curative resection (*i.e.*, R0 margins) of hilar CC, which range from 27% to 45% in selected cases (reviewed by [4]). However, a comparison of the different trials published so far is difficult because many studies do not separately indicate survival rates for distinct tumor stages, and patient survival after curative resection of hilar CC strongly correlated with the proportion of patients without metastases (lymph node or distant, reviewed in [6]). Therefore, we compared the long-term outcome after neoadjuvant PDT with that of our patients with non-pretreated hilar CC that had undergone R0 resection by the same surgeons during that time [20].

Tumor stages of both groups were nearly comparable. Of note, more patients were lymph node-positive (yet not significant) and Ca 19-9 serum concentration was higher in spite of less cholestasis—factors biasing for increased risk of CC recurrence in the neoadjuvant series. Nevertheless, the survival curves of both trials compared favorably for seven years, with a tendency (not significant) for longer survival rates in the neoadjuvant group, and was significantly better than the survival of the historical cohort resected R1 (figure 2 in [20]). However, the actual pilot series contains a very small number of patients, and the survival data are only explorative.

Available data on neoadjuvant therapy in hilar CC is still rare. Since there is currently no evidence to suggest an improved survival outcome with neoadjuvant therapy in *resectable* patients (systematically reviewed in [30]), the main goal of this concept is to achieve resectability in patients staged *non-resectable* due to slightly advanced tumor extension in the bile duct branches/segments (or as a bridging strategy during the waiting time before OLT in highly selected patients). The current pilot study showed a histologically proven 100% local response, *i.e.*, complete tumoricidal effect of PDT, within the 4 mm deep inner periluminal layer of hilar CC [27]. Since the local response rate in studies investigating chemotherapy in the context of hilar CC is below 30%, this would restrict the benefit of a neoadjuvant chemotherapeutic strategy (reviewed in [30,31]). Some studies have demonstrated encouraging outcomes after neoadjuvant chemoradiation in the context of OLT [4,31,32,33]. However, in the context of surgical resection after neoadjuvant chemoradiotherapy with or without brachytherapy, only case series or retrospective reviews have been published so far. McMasters *et al.* [34] described nine patients with extrahepatic cholangiocarcinoma (including 5 hilar CC) considered unresectable and downstaged by external beam radiotherapy, resulting in a 100% R0 resection rate. Another cohort of 12 patients with unresectable hilar and distal CC underwent chemoradiation (±brachytherapy), leading to a R0 resection rate of 91% and a 5-year survival rate of 53% [35]. 

The tumor selectivity and high rate of local response at a low rate of tolerable adverse events would favor PDT over chemoradiation as neoadjuvant therapy, especially when performed with a deeper tumoricidal effect, as shown for temoporfin [24,25] and talaporfin [23]. Concerning the high rates of distant failure, *i.e.*, distant metastatic recurrence, probably more effective systemic therapies are required as (neo-)adjuvant treatment. Of note, Nonaka *et al.* demonstrated that PDT combined with oxaliplatin and gemcitabine, showed significantly higher local cytotoxic effects in comparison with PDT alone in tumor xenografts in mice [36]. In a phase II randomized study, Park *et al.* could show a significant survival benefit, if PDT was combined with S-1 chemotherapy in patients with non-resectable hilar CC [37]. The promising strategy of combined systemic and local ablating therapy is currently under investigation (PDT using Photofrin^®^ and gemcitabine/cisplatin *vs.* chemotherapy alone, phase III randomized clinical trial, “Pinnacle-PHO1201”; NCT02082522 [38]).

## 3. Experimental Section

### Statistics

Data were summarized as median and range, or as mean values with standard error of the mean (SEM) where appropriate. Patient survival was calculated with the Kaplan-Meier method. Survival data of the actual study and the historical cohort of 35 patients resected R0 without pre-treatment in a previous trial [20] were compared in a retrospective manner using the log rank test. Furthermore, comparisons of categorical and continuous variables were performed with the Mann-Whitney’s *U* test. Results obtained by this comparison are referred to as “compared to the historical cohort”. Differences were considered statistically significant if the *p*-value was less than 0.05. Analyses were performed using the statistical package SPSS version 17.0 (IBM, Vienna, Austria).

## 4. Conclusions

Based on the current long-term outcome analysis, down-sizing tumor margins of hilar CC with neoadjuvant PDT is feasible and appears to achieve R0 resection that could improve overall survival of patients considered non-curatively resectable because of tumor extension along the bile duct branches. However, neoadjuvant PDT prior to surgery needs to be validated against surgery only in a randomized trial for Bismuth type III and IV cholangiocarcinomas staged borderline non-resectable because of extent of tumor along bile duct branches/segments. In such a trial, a photosensitizer with deeper tumoricidal tissue penetration should be used—preferably combined with systemic therapy for prevention of distant failure.
